# Omega-3 Polyunsaturated Fatty Acids Prevent Nonalcoholic Steatohepatitis (NASH) and Stimulate Adipogenesis

**DOI:** 10.3390/nu13020622

**Published:** 2021-02-15

**Authors:** Vitor Jacó Antraco, Bruna Kelly Sousa Hirata, Jussara de Jesus Simão, Maysa Mariana Cruz, Viviane Simões da Silva, Roberta Dourado Cavalcante da Cunha de Sá, Fernanda Miranda Abdala, Lucia Armelin-Correa, Maria Isabel Cardoso Alonso-Vale

**Affiliations:** Department of Biological Sciences, Institute of Environmental Sciences, Chemical and Pharmaceutical, Universidade Federal de São Paulo—UNIFESP, Diadema 09913-130, Brazil; vitor_antraco@hotmail.com (V.J.A.); bruhirata@gmail.com (B.K.S.H.); jus_simao@hotmail.com (J.d.J.S.); maysamariana@gmail.com (M.M.C.); ss.vivi95@gmail.com (V.S.d.S.); rdccunha@gmail.com (R.D.C.d.C.d.S.); fernanda.abdala03@gmail.com (F.M.A.); larmelincorrea@gmail.com (L.A.-C.)

**Keywords:** obesity, fish oil, beige adipocytes, liver, AdScs

## Abstract

The increasing impact of obesity on global human health intensifies the importance of studies focusing on agents interfering with the metabolism and remodeling not only of the white adipose tissue (WAT) but also of the liver. In the present study, we have addressed the impact of n-3 PUFA in adipose cells’ proliferation and adipogenesis, as well as in the hepatic lipid profile and morphology. Mice were induced to obesity by the consumption of a high-fat diet (HFD) for 16 weeks. At the 9th week, the treatment with fish oil (FO) was initiated and maintained until the end of the period. The FO treatment reduced the animals’ body mass, plasma lipids, glucose, plasma transaminases, liver mass, triacylglycerol, and cholesterol liver content when compared to animals consuming only HFD. FO also decreased the inguinal (ing) WAT mass, reduced adipocyte volume, increased adipose cellularity (hyperplasia), and increased the proliferation of adipose-derived stromal cells (AdSCs) which corroborates the increment in the proliferation of 3T3-L1 pre-adipocytes or AdSCs treated in vitro with n-3 PUFA. After submitting the in vitro treated (n-3 PUFA) cells, 3T3-L1 and AdSCs, to an adipogenic cocktail, there was an increase in the mRNA expression of adipogenic transcriptional factors and other late adipocyte markers, as well as an increase in lipid accumulation when compared to not treated cells. Finally, the expression of browning-related genes was also higher in the n-3 PUFA treated group. We conclude that n-3 PUFA exerts an attenuating effect on body mass, dyslipidemia, and hepatic steatosis induced by HFD. FO treatment led to decreasing adiposity and adipocyte hypertrophy in ingWAT while increasing hyperplasia. Data suggest that FO treatment might induce recruitment (by increased proliferation and differentiation) of new adipocytes (white and/or beige) to the ingWAT, which is fundamental for the healthy expansion of WAT.

## 1. Introduction

Obesity is a chronic disease defined as an imbalance between energy intake and calorie expenditure, with an abnormal or excessive lipid accumulation in adipose tissue and other organs (e.g., liver, pancreas, and skeletal muscle) [1,2]. Increased adipose mass is directly associated with chronic low-grade systemic inflammation that is involved in the development of insulin resistance, type 2 diabetes mellitus (T2DM), cardiovascular diseases, nonalcoholic fatty liver disease (NAFLD), and metabolic syndrome. These comorbidities highlight the crucial role of white adipose tissue (WAT) in body homeostasis [2,3].

The overload of triglycerides (TAG) in the adipose tissue induces a pro-inflammatory profile, due to adipocytes hypertrophy, WAT hypoxia, macrophage recruitment and activation, and dysregulation of adipokine secretory patterns [4]. The insulin resistance also leads to weaker insulin antilipolytic effect and fatty acid metabolism resulting in an increased release of free fatty acids (FFA) [5]. The elevated hepatic influx of lipids, including FFA, TGs, free cholesterol, and ceramides, are related to hepatic apoptosis, which is a key feature of nonalcoholic fatty liver disease (NAFLD) [6]. In addition, cytokines such as leptin, resistin, angiotensinogen, TNF-α, and interleukins also appear to play a notable role in the development of the disease [7].

Overweight and obesity are characterized as excessive fat accumulation and are major risks for liver complications [8,9]. NAFLD encompasses a spectrum of liver diseases which are at the first stage characterized by the exacerbated lipids accumulation into the hepatocytes. The progressive form is nonalcoholic steatohepatitis (NASH) characterized by hepatocyte damage due to inflammation, ballooning, and fibrosis by collagen deposition, which can worsen over time and may lead to cirrhosis and hepatocellular carcinoma (HCC) [10]. NAFLD is highly associated with obesity and insulin resistance. The overall obesity prevalence estimates among NAFLD and NASH patients are 51% and 82%, respectively, while T2DM was identified in 23% of NAFLD cases and 44% of NASH cases [11]. 

The multifactorial mechanisms driving from NAFLD to NASH include oxidative stress, lipotoxicity, and mitochondrial damage [12]. The incapacity of hepatocytes to dispose of excess FFAs results in apoptosis by intracellular stresses and by organelle dysfunction, including endoplasmic reticulum (ER) stress and mitochondrial permeabilization [13]. Plasma markers of liver injury can also be detected such as alanine aminotransferase (ALT), aspartate aminotransferase (AST), alkaline phosphatase, and gamma-glutamyl transpeptidase (GGT).

Beforehand, we described that fish oil (FO), which is rich in n-3 long-chain polyunsaturated fatty acids (n-3 PUFA), mainly eicosapentaenoic acid (EPA, 20:5 n-3) and docosahexaenoic acid (DHA, 22:6 n-3), had positive effects on preventing obesity and its deleterious effects. In our previous studies, FO administration was either preventive, beginning four weeks before the induction of obesity [14], or as a treatment after the induction of obesity, FO was administered to the animals only eight weeks after the induction of obesity, and administration was maintained for another eight weeks [15]. PUFA-rich diets (as Mediterranean diet with high consumption of nuts and fish) have been described as a helpful instrument in preventive medicine, attenuating NAFLD/NASH by decreasing liver fat content, improving insulin sensitivity, and lipid profile [16,17,18]. However, it remains unclear how n-3 PUFA can act remodeling adipose tissue and attenuating NAFLD. Herein, using our previous protocol of obesity treatment with FO, we aimed to investigate if n-3 PUFA can affect mesenchymal adipose-derived stromal cells (AdSC) inducing adipogenesis in WAT and if it has beneficial effects on reversing liver steatosis.

## 2. Materials and Methods

### 2.1. Animals and Fish Oil Supplementation

The study was performed according to protocols approved by the Ethics Committee of the Federal University of São Paulo (CEUA 2008220218). Eight-week-old male C57BL/6 mice obtained from the Center for Development of Experimental Models (CEDEME), Federal University of São Paulo (UNIFESP), were housed in a room with a light-dark cycle of 12–12 h and temperature of 24 ± 1 °C. The experimental protocol remained for 16 weeks, where, in the first 8 weeks (first period), mice were separated into two groups: control (CO, 9% fat, 76% carbohydrates, and 15% proteins) and high-fat diet (HFD, 26% carbohydrates, 59% fat, and 15% proteins) group. We have previously demonstrated that this duration and composition of HFD results in obesity, glucose, and insulin intolerance, increased fasting blood glucose and insulin levels, raised blood cholesterol and low-density lipoproteins (LDL) cholesterol concentrations, and elevated homeostatic model assessment of insulin resistance (HOMA-IR), thus characterizing the metabolic syndrome [14,15]. In the next 8 weeks (second period), the HFD group was subdivided into HFD and HFD + FO (high-fat diet supplemented with FO) groups. Supplementation was performed three times per week, by oral gavage at 2 g/kg BW (n-3 PUFA source, 5:1 EPA/DHA ratio, HiOmega-3, Naturalis Nutrição, and Farma Ltda., São Paulo, Brazil). The CO and HFD groups received water by gavage at the same volume (~50 μL, according to the BW). The FO dosage was chosen based on previous studies from our group [14]. Considering the body surface area for extrapolating the dose used in the present study to humans [19], FO 2 g/kg BW in mice is ~162 mg/kg BW in humans, that is, ~11 g in a person with 70 kg, every two days. Human studies reported a dose of 6 g of FO every day [20,21]. Therefore, the dosage used here may be equivalent to human studies.

### 2.2. Experimental Procedure

Bodyweight and food intake were measured once a week. After 16 weeks of the experimental protocol, 10–12 h fasted mice were anesthetized with isoflurane and euthanatized by cervical dislocation after blood collection through puncturing the orbital plexus. Blood samples were centrifuged at 1500 rpm for 20 min at 4 °C and serum was stored at −80 °C. The subcutaneous adipose fat depot (inguinal—ingWAT) was harvested, weighed, and processed as described below.

### 2.3. Biochemical Plasma and Lipid Analysis in the Liver

Triacylglycerol [22], total cholesterol, LDL-cholesterol [23], and HDL-cholesterol levels [24] were determined by colorimetric assays (Labtest Diagnostics, Lagoa Santa, MG, Brazil). Total cholesterol, HDL cholesterol, LDL cholesterol, bilirubin, aminotransferase (ALT), aspartate aminotransferase (AST), alkaline phosphatase and gamma-glutamyltransferase (GGT), triglycerides, and glycemic were measured through a colorimetric enzyme kit (Labtest, Lagoa Santa, MG, Brazil) and non-esterified fatty acid (NEFA) was measured using the NEFA RH series kit (Wako Chemicals Inc., Richmond, VA, USA) according to the manufacturer’s instructions. Fragments of the liver tissue were also removed from the animals (as described below) for the quantification of triglycerides and total cholesterol.

### 2.4. Frozen Liver Tissue H/E and Oil Red O Staining

Paraffin sections 10 μm thick were obtained from frozen liver tissue and then subjected to the staining process with hematoxylin and eosin according to protocols described by Bancroft; Gamble [25], Fischer et al. [26], and Catta-Preta et al. [27] to assess the morphology of hepatocytes, as well as oil red O to observe the presence of lipid drops. The tissue sections were observed and photographed under an optical microscope (20× and 40×) connected to the camera (AxioCam ERc5s; Zeiss, Oberkochen, Germany).

### 2.5. Adipocytes and AdSC Isolation

Subcutaneous ingWAT was removed, weighed, and placed in a digestion buffer. Cells went through the digestion of the tissue by collagenase [28] with some modifications. Briefly, the fat pad was digested in Dulbecco’s Modified Eagle Medium (DMEM) supplemented with HEPES (20 mM), sodium pyruvate (2 mM), bovine serum albumin (BSA, 1%), and collagenase type II (1 mg/mL), pH 7.4 at 37 °C in an orbital bath shaker for about 45 of 60 min. After elimination by filtration through a nylon mesh (Corning, NY, USA) of undigested fragments, the filtrate was centrifuged (400× *g*, 1 min), and then divided into two fractions: 1. The floating adipocyte layer, and 2. The stromal-vascular fraction (SVF) cells (all remaining filtrate), which was subjected to centrifugation (1500× *g* for 10 min). Cells were pooled from two mice. One pooled cell was counted as one sample. Isolated mature adipocytes were washed three times in fresh buffer without collagenase. After washing and brief spin, the medium was thoroughly aspirated, and adipocytes were harvested. Aliquots of isolated adipocytes suspensions were placed in a microscope slide and 6 fields were photographed under an optical microscope (×100 magnification) coupled to a microscope camera (AxioCam ERc5s; Zeiss, Oberkochen, Germany), and mean adipocyte volume (4/3 × π × r3) was determined by measuring 100 cells using AxioVision LE64 software. 

### 2.6. AdSC Culture and Differentiation 

The cellular pellet containing the SVF was resuspended in a pre-warmed incubation medium [D’MEM F-12, supplemented with 10% bovine fetal serum (FBS) and 10 mL/L penicillin/streptomycin (Gibco BRL, Grand Island, NY, USA)], seeded in 10 cm plates. After 2 h, the medium was changed to remove red blood cells and other residues. Cells were cultured in 5% CO_2_ at 37 °C until reaching 70–80% of confluency (2 passages). The final step for the isolation of AdSCs is the selection of the adherent population within the SVF. The cells were finally plated at a density of 1 × 10^5^ cells/well in a 96-well plate (for proliferation analysis) or 1 × 10^6^ cells/well in a 6-well plate for other analysis. To induce adipogenesis, 2 days after confluence (day 0), cells were stimulated by the differentiation induction medium composed of 0.5 mM IBMX (3-isobutyl-1-methylxanthine), 1 µM dexamethasone, and 1.67 µM insulin, supplemented with 50 nM triiodothyronine, for 2 days. The adipocyte maturation medium consisted of D’MEM F-12 (10% FBS), 0.4 µM insulin, and 50 nM triiodothyronine, and was changed every 2 days. After 6 days of induction of differentiation, adipogenesis was estimated by the accumulation of lipids, through oil red O staining.

### 2.7. In Vitro Treatment with Fatty Acids

The n-3 PUFA EPA and DHA obtained from Sigma, were dissolved in ethanol (vehicle) not exceeding 0.05% and added to the cells during the proliferation (MTT assays) or at day 0 with the differentiation induction medium and maintained until the end of the respective assay. For the in vitro treatments, cells received EPA or DHA at a concentration of 50 μM, alone or combined at a 5:1 ratio (42 μM EPA: 8 μM DHA, the total concentration of 50 μM), simulating the fatty acid composition of the FO used for the animals from this study. 

### 2.8. Differentiation of Pre-Adipocytes from 3T3-L1 Cell Lineage and Browning Induction

Pre-adipocytes cloned from disaggregated mice embryos (Swiss 3T3 cells) obtained from the cell bank of Rio de Janeiro (RJ, Brazil) were also cultivated and maintained in D’MEM supplemented with 10% of calf serum (CS) and kept in a 5% CO_2_ incubator at 37 °C until they reached the confluence (day-2). Differentiation induction medium (containing D’MEM instead of D’MEM F-12) was used to stimulate adipogenesis of 3T3-L1 cells at day 0, for two days. Then, the preadipocytes were cultured in an adipocyte maturation medium (without triiodothyronine) for 4 additional days, being changed every 2 days.

To stimulate browning in adipocytes, the differentiation medium was supplemented with 50 nM triiodothyronine and 1 μM rosiglitazone (Sigma, St. Louis, MI, USA) cocktail for browning [29] in the presence or absence of the respective PUFAs for 2 days, and, then, cultured in adipocyte maturation medium for 6 additional days.

### 2.9. Cell Proliferation Assay

Cell proliferation (which is directly proportional to the number of viable cells in culture) was evaluated by the 3-[4,5-dimethylthiazol-2-diphenyltetrazolio] (MTT) formazan bromide reduction method by the MTT cell proliferation Kit (Cat. No. 11465007001, Roche Diagnostics, Mannheim, Germany) [30]. Briefly, after 36 h of culture (5 × 10^3^ cells/well at 100 μL D’MEM F-12/FBS) in 96-well plates (flat bottom), the cells were added at 100μL/well of MTT and incubated for 4 h (37 °C, 5% CO_2_); after this period, the formazan crystals’ solubilization solution (10% SDS at HC l0.01M) was added at 10μL/well. The plates were then incubated for 14 h (37 °C, 5% CO_2_). After incubation, the absorbance (at 550 nm) was measured in an optical plate reader. Since all cultures were plated with the same initial number of cells, an increase or decrease of viable cells represent the potential for proliferation of pre-adipocytes, which was expressed as percentage values concerning the control.

### 2.10. Oil Red O Staining and Lipid Content Determination

Six days after differentiation induction, the cells were washed twice with PBS and fixed with 10% formalin in PBS and stained in 0.3% oil red O (Sigma) solution in 60% (*v*/*v*) 2-propanol in water for 1 h, for marking and visualization of lipid content. The cells were photographed, and the lipid content was analyzed by spectrophotometry.

### 2.11. RNA Extraction and Quantitative Real-Time Polymerase Chain Reaction (qPCR)

Total RNA from 3T3-L1 cell lysates was extracted using Trizol (Invitrogen Life Technologies, Carlsbad, CA, USA), analyzed for quality on an agarose gel and absorbance ratios of 260/280 nm and 260/230 nm, and reverse transcribed to cDNA using the SuperScript III cDNA kit (Invitrogen Life Technologies). Gene expression was evaluated by real-time qRT-PCR using a Rotor-Gene (Qiagen, Roermond, The Netherlands) and SYBR Green as a fluorescent dye (Qiagen) with *36B4* as a housekeeping gene. The reaction conditions were as follows: 95 °C for 5 min, then 40 cycles of 95 °C for 5 s, and 60 °C for 10 s. PCR products were run on an agarose gel to confirm the size of the fragment and specificity of amplification. The primers were designed and will be used to quantify the messenger RNA (mRNA) encoded by the genes listed in Table 1. Data were obtained as ct values (ct = cycle number at which logarithmic PCR plots cross a calculated threshold line) and used to determine ∆ct values (∆ct = (ct of the target gene) − (ct of the housekeeping gene). Data were expressed as arbitrary units using the following calculation: [expression = 1000 × (2 − Δct) arbitrary units (AU)].

### 2.12. Statistical Analysis

Data are expressed as mean ± standard error of the mean (SEM). A student’s *t*-test was used in the first period, and one-way ANOVA variance analysis, followed by Tukey’s post-test, in the second period for comparisons between groups. Prism, version 5.0 (GraphPad Software, Inc., San Diego, CA, USA), was used for analysis. *p* < 0.05 was considered statistically significant.

## 3. Results

### 3.1. Effects of FO Treatment on Body Weight, Adiposity, and Hypertrophy of ING Adipocytes from Mice with HFD-Induced Obesity

**Figure 1:**
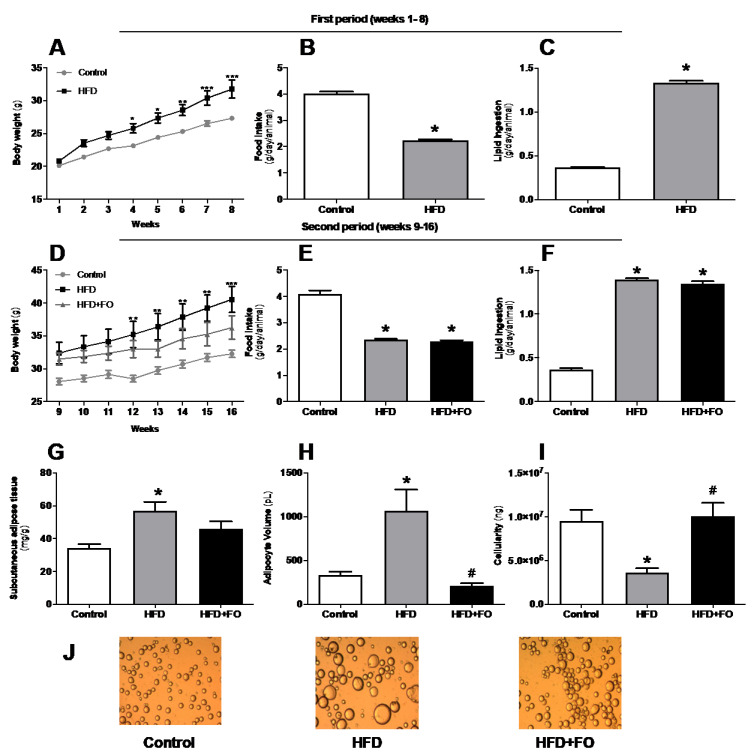
Effects of high-fat diet (HFD) and fish oil (FO) treatment on body weight, food intake, and adiposity. In the first period, mice were fed with a control diet (control group) or HFD. In the second period, the diets were maintained and water (CO and HFD groups) or fish oil (HFD + FO group) were delivered by orogastric gavage. (**A**,**D**) body weight (g); (**B**,**E**) food intake (g/day/animal); (**C**,**F**) lipid ingestion (g/day/animal); (**G**) subcutaneous adipose tissue (mg/g), (**H**) ingWAT adipocyte volume (pL); (**I**) ING cellularity (ng); (**J**) isolated ING adipocytes photographed under optic microscope (×100 magnification). Results were analyzed by one-way ANOVA and Tukey post-test. Values are mean ± SEM (n = 10–20). * *p* < 0.05.

The evolution of the animals’ body mass (in grams) over 16 weeks is shown in Figure 1. In the period I (weeks 1–8), animals fed with HFD, which is rich in saturated fat, gained 30% more body mass than animals fed with a CO diet (Figure 1A). In period II (weeks 9–16), we observed that the animals receiving HFD (both HFD and HFD + FO groups) presented an increase in body mass of 48% and 29%, respectively, when compared to the CO group (Figure 1D). Thus, treatment with FO partially reversed the body mass gain, promoting a reduction of 27% when compared to the HFD group (Figure 1D). 

The animals’ food intake was measured (twice a week) and the analysis of the results regarding the consumption is also shown in Figure 1. The animals were given a CO or HF diet for a total period of 16 weeks. In the first 8 weeks (period I), the animals only received the control or high-fat diet. It was possible to observe a decrease in food intake (44%, Figure 1B) by the HFD group compared to the CO group. However, there was a higher lipid intake (72%, Figure 1C) by the HFD group when compared to CO. In period II (weeks 9–16), the introduction of FO did not change these feeding patterns, and the differences between groups observed in period I was maintained on the same parameters (Figure 1E,F). 

IngWAT was removed and the relative mass (mg/g body mass) of this cushion was calculated. It was found that there was a significant increase (40%) in the deposit mass of the HFD group when compared to the CO group. However, supplementation with FO partially reversed this increase, causing a 20% reduction in the HFD + FO compared to the HFD group (Figure 1G).

Likewise, it was observed that the HFD group presented significant hypertrophy of the ingWAT adipose cells (65% increase) when compared to the CO group (Figure 1H). The FO supplementation completely prevented this effect, since it was observed a 74% adipocyte volume reduction in comparison to the HFD group (Figure 1H,J). In addition, FO significantly increased by 60% the tissue cellularity (Figure 1I), which may be a strong indication that the treatment is favoring hyperplasia or adipogenesis in this fat depot.

### 3.2. Plasma Lipid Profile and Blood Glucose

**Figure 2:**
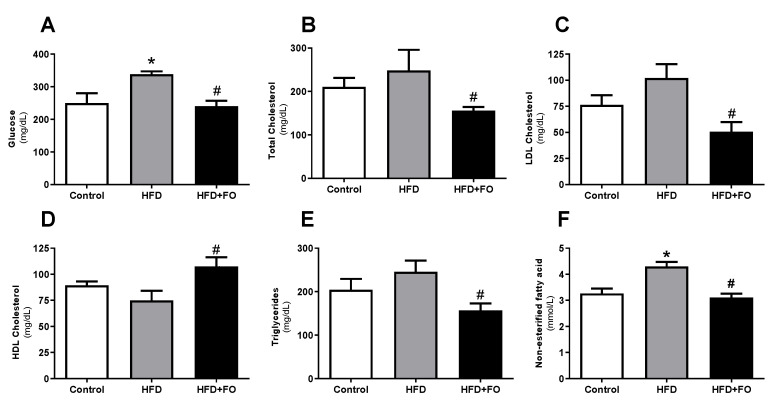
Plasma biochemical profile: (**A**) fasting glucose (mg/dL), (**B**) total cholesterol (mg/dL), (**C**) LDL cholesterol (mg/dL), (**D**) HDL cholesterol (mg/dL), (**E**) triglycerides (mg/dL), and (**F**) NEFA (mmol/L), in animals with diet-induced obesity for 16 weeks. The animals received control diet (Control group) or hyperlipidic diet without (HFD group) or with fish oil supplementation in the last eight weeks (HFD + FO group). Values expressed as mean ± SEM (n = 10–20). * *p* < 0.05 vs. Control; # *p* < 0.01 vs. HFD; one-way ANOVA and Tukey post-test.

As shown in Figure 2, HFD induced an increase in fasting plasma glucose (26%, Figure 2A), total cholesterol (15%, Figure 2B), LDL-c (25%, Figure 2C), triglycerides (16%, Figure 2E), and NEFA (24%, Figure 2F), as well as reduced HDL-c (16%, Figure 2D) in the HFD group when compared to the CO group. However, FO treatment showed a positive effect on reduction of fasting glucose (29%), total cholesterol (37%), LDL-c (50%), triglycerides (36%), and NEFA (27%) when compared to HFD. HFD + FO group also had a 30% increase in HDL-c.

### 3.3. Effects of a High-Fat Diet and Treatment with Fish Oil on Nonalcoholic Steatohepatitis (NASH)

**Figure 3:**
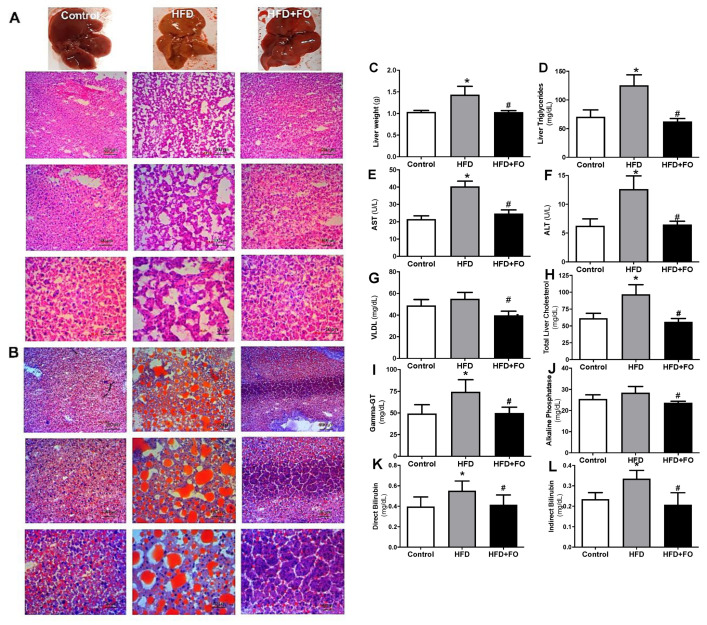
Macroscopic and histological comparison after (**A**) H&E and (**B**) oil red O staining for visualization of hepatic lipid accumulation; (**C**) absolute weight of the liver (g); (**D**) liver triglycerides (mg/dL); (**E**) plasma AST (U/L); (**F**) plasma ALT (U/L); (**G**) VLDL-c (mg/dL); (**H**) liver total cholesterol (mg/dL); (**I**), plasma Gamma-GT (mg/dL); (**J**) plasma alkaline phosphatase (mg/dL); (**K**) direct bilirubin (mg/dL); (**L**) indirect bilirubin (mg/dL). Mice received control diet (Control) or hyperlipidic diet without (HFD) or with fish oil treatment (HFD + FO). Values expressed as mean ± SEM (n = 10–20). * *p* < 0.05 vs. Control, # *p* < 0.05 vs. HFD; one-way ANOVA and Tukey post-test.

Histological sections of the liver were performed to compare biochemical measurements. In fact, by microscopic analysis with H&E (Figure 3A) or oil red O staining (Figure 3B), the morphology of hepatocytes and the presence of lipid drops were confirmed in the HFD group, while microgoticular hepatocytes were observed in control animals. 

In obese animals fed with HFD for 16 weeks, it can be observed diffuse macrogoticular hepatocytes, with abnormal deposition of TAG in the parenchymal cells, characterized by the presence of clear vacuoles in the cytoplasm around the cell nucleus. The affected organ increased in size and presented a yellowish aspect, which perhaps may be associated with loss of function and with worsening of a pathological condition. Interestingly, protection/reversion of this condition in animals that received FO was observed, given the reduced lipid deposition in the hepatic histology section of this group. 

It was observed that, when compared to the CO group, the animals that received HFD showed a NASH condition since there was an increase in the total liver mass (28%, Figure 3C), liver triglycerides (43%, Figure 3D), liver transaminases: AST (50%, Figure 3E) and ALT (47%, Figure 3F), VLDL (11%, Figure 3G), total liver cholesterol (36%, Figure 3H), Gamma-GT (34%, Figure 3I), alkaline phosphatase (11%, Figure 3J), direct bilirubin (29%, Figure 3K) and indirect bilirubin (30%, Figure 3L). The treatment with FO was able to reverse this situation, reducing all these parameters in comparison to the HFD group (~ 28%, 50%, 49%, 39%, 30%, 42%, 32%, 17%, 25%, and 39%, respectively).

### 3.4. Effects of FO Treatment on Proliferation and Differentiation of AdSCs Isolated from Obese Mice 

**Figure 4:**
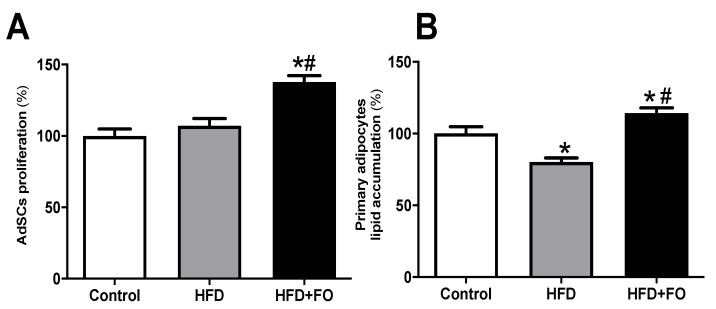
AdSCs proliferation and adipocyte differentiation. (**A**) the proliferation and (**B**) evaluation of intracellular lipid accumulation determined after oil red O staining and estimated by spectrophotometry after induction of adipogenesis of primary pre-adipocytes from control diet (Control) or hyperlipid diet (HFD) mice treated with fish oil (HFD + FO). The pre-adipocytes were obtained from the cell fraction of the vascular stroma of the WAT inguinal. Values expressed as mean ± SEM (n = 6–15). * *p* < 0.001 vs. Control, # *p* < 0.01 vs. HFD; one-way ANOVA and Tukey post-test.

Evaluating the effect of in vivo treatment with FO on the proliferation of primary mouse pre-adipocytes, we observed that the proliferation of AdSCs isolated from the HFD + FO group was higher than CO and HFD groups, 27% and 22%, respectively (Figure 4A). 

The adipogenic potential of AdSC after adding the differentiation stimulus (adipogenic cocktail) was estimated using oil red O staining and spectrophotometry measurement. The adipocytes differentiated from HFD mice AdSCs showed a 20% reduction in the lipid content when compared to the CO group (Figure 4B). The FO treatment completely prevented this deleterious effect and had a positive impact on the cell ability to accumulate lipids after in vitro differentiation (42%), suggesting an important role of FO treatment on AdSCs adipogenic potential (Figure 4B).

### 3.5. Effects of In Vitro n-3 PUFA Treatment on Proliferation, Differentiation, and Gene Expression of Markers of Adipogenesis on Both AdSCs and 3T3-L1 Pre-Adipocytes

**Figure 5:**
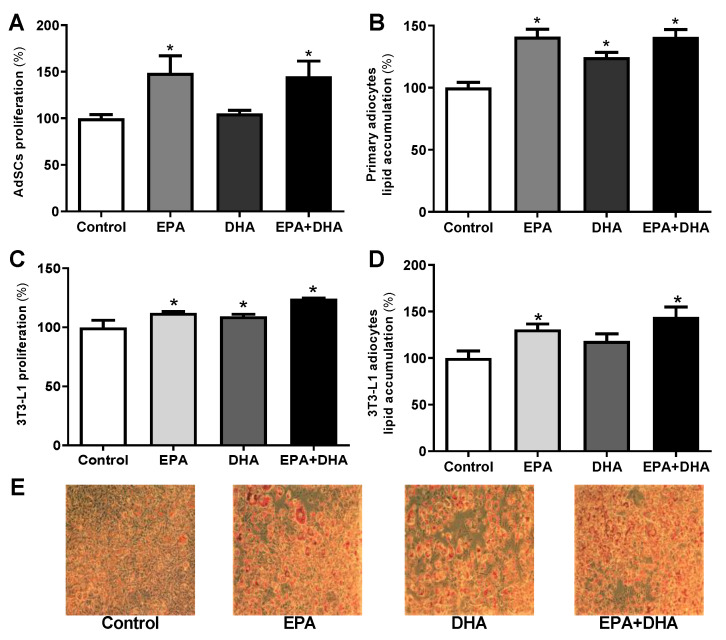
Proliferation, evaluation of cell lipid accumulation determined by oil red O staining after induction of adipogenesis in primary mice pre-adipocytes (**A**,**B**) and 3T3-L1 (**C**–**E**) cultivated in vitro in the presence or not (control) of 50 µM of eicosapentaenoic acid (EPA, C20:5 n-3), docosahexaenoic (DHA, C22:6 n-3), its association (EPA/DHA) in a 5:1 ratio (42 µM EPA: 8 µM DHA). Values expressed as mean ± SEM (n = 6–15). * *p* < 0.05 vs. Control; one-way ANOVA and Tukey post-test.

When we cultivated primary pre-adipocytes from eutrophic mice (not fed with HFD or HFD + FO) with n-3 PUFA (EPA and DHA alone, or associated in a 5: 1 ratio), we observed an increase of 33% (treatment with EPA) and 31% (treatment with EPA/DHA association) in proliferation potential compared to control. This effect was not seen in the presence of DHA (Figure 5A).

The in vitro treatment of AdSCs extracted from eutrophic mice (from day 0 of differentiation induction) with omega-3 [eicosapentaenoic acid (EPA, C20:5 n-3), docosahexaenoic (DHA, C22:6 n-3) and its association (EPA/DHA)] promoted an increase in lipid content of 30%, 20%, and 30%, respectively, as we can see in Figure 5B.

We also evaluated the isolated and combined EPA and DHA treatment on 3T3-L1 preadipocytes. 3T3-L1 preadipocytes treated for 36 h with EPA, DHA, or their association (EPA/DHA) showed an increase in proliferation potential of 11%, 9%, and 20%, respectively, when compared to the control group (Figure 5C). The treatment with EPA (alone or in association with DHA), also increased the lipid accumulation by 24% and 31%, respectively (Figure 5D). These effects were visualized by microscopy after staining (Figure 5E).

Several transcription factors act on the adipogenesis process. Peroxisome Proliferator-Activated Receptor gamma (*Pparγ*) and CCAAT Enhancer Binding Protein Alpha (*Cebpα*) have a key role in the metabolism of adipose tissue and directly affect the differentiation of pre-adipocytes, being known as the “master” regulators of adipogenesis (Rosen & MacDougald, 2006). The use of the 3T3-L1 lineage is of extreme importance to elucidate this process. The expression of the genes that encode these transcriptional factors and other genes involved in the process of adipocytes differentiation were analyzed. 

**Figure 6:**
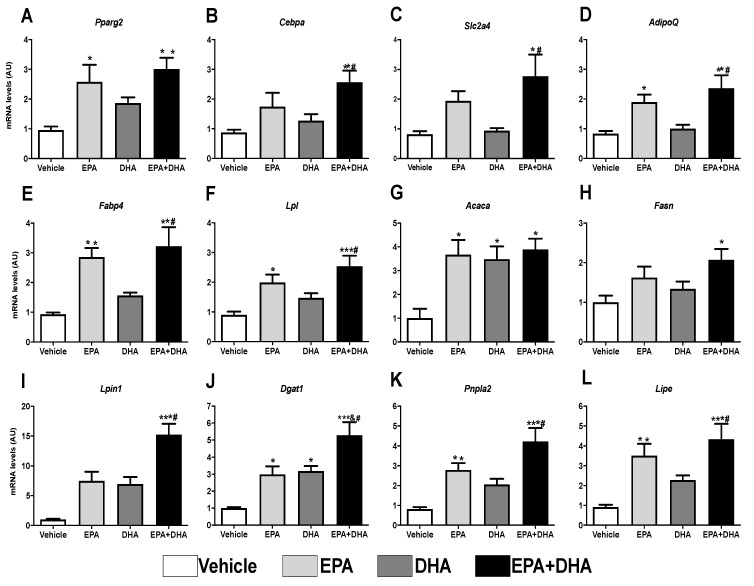
Evaluation of gene expression 8 days after differentiation of 3T3-L1 preadipocytes by RT-PCR in the presence or not (control) of 50 µM of eicosapentaenoic acids (EPA, C20:5 n-3), docosaexaenoic (DHA, C22:6 n-3) or their association in a 5:1 ratio (42 µM EPA: 8 µM DHA). (**A**) *Ppar-gamma*; (**B**) *C/ebp-alpha*; (**C**) *Sic2a4* (Glut-4); (**D**) *AdipoQ* (Adiponectin); (**E**) *Fabp4*; (**F**) *Lpl*; (**G**) *Acaca* (Acc1); (**H**) *Fasn* (FAS); (**I**) *Lpin1* (Lipin); (**J**) *Dgat1*; (**K**) *Pnpl2* (Atgl); (**L**) *Lipe* (Hsl). Values of mRNA were expressed in relation to the control and corrected by the expression of the constitutive gene *36B4*. Values are expressed as mean ± SEM (n = 4–7). * *p* < 0.05 vs. Control, ** *p* < 0.01 vs. Control, *** *p* < 0.001 vs. Control, # *p* < 0.01 vs. DHA. One-way ANOVA and Tukey post-test.

We observed that, after 8 days of induction with the adipogenic cocktail, the presence of 50 µM of EPA or its association with DHA (EPA/DHA) since the day of induction (day 0) promoted an increase in the expression of *Pparγ* (62% and 68%, respectively) and *Cebpα* (50% and 65%, respectively) when compared to the control group (adipogenic cocktail alone) (Figure 6A,B). 

Corroborating the greater expression of adipogenic transcriptional factors, treatment with EPA (alone or in association with DHA) also increased the gene expression of markers that respond to *Pparγ* and *Cebpα*: the *Glut4* glucose carrier, 58%, and 71%, respectively (Figure 6C) and *Adiponectin*, 56% and 65%, respectively (Figure 6D).

The results also revealed that, when compared to the control group, EPA, as well as its association with DHA (EPA/DHA), promoted an increase of 67% and 71%, respectively, in the expression of the gene that encodes *Fabp4*, a protein that is involved in the adipocyte binding and intracellular transport of fatty acids (Figure 6E). EPA and EPA/DHA also led to a significant increase in the expression of genes that encode lipogenesis-related proteins, such as *LPL*, which promotes the uptake of free fatty acids (55% and 64%, respectively), of *Acc* (~74%) and *Fas* (38% and 52%, respectively), both involved with the synthesis of fatty acids again, besides *Lpin* (33% and 93%, respectively) and *Dgat1* (67% and 81%, respectively), the latter two involved in the synthesis of TAG (Figure 6E–J). 

In the same way, concerning the genes that encode proteins that perform lipolysis, we also observed an increase in the expression of *Atgl* (70% and 81%) and *Hsl* (74% and 79%), in the cells treated in vitro with EPA, alone or associated to DHA (EPA/DHA), respectively (Figure 6K,L). Interestingly, DHA fatty acid did not show any effect concerning these parameters.

The increased expression of the adipogenic transcriptional factors and the adipocyte terminal differentiation markers agree with the results of lipid content estimated in the cells treated with these PUFAs. EPA treatment (alone or associated with DHA) boosted lipid accumulation (Figure 5D). Once more, these effects were not observed in the presence of DHA only. Taken together, these data suggest that EPA stimulates adipocyte proliferation and differentiation.

### 3.6. Analysis of Gene Expression of Beige Adipocyte Markers after Browning Induction in 3T3-L1 Cells

The following analyses of the expression of genes involved in the oxidation of fatty acids (*Cpt1*), mitochondrial function (*Nrf1* and *Tfam*), thermogenesis, and mitochondrial biogenesis (*Ucp1*, *Pgc1*-*α, Prdm16, Cited, Cidea, Tmem 26, Tbx1, Fgf21, Dio2,* and *Bmp7*) are demonstrated in preadipocytes of cell line 3T3-L1 in the presence, or not (control), of 50 µM of EPA, DHA, or their association in a ratio 5: 1 (42 µM EPA: 8 µM DHA).

**Figure 7:**
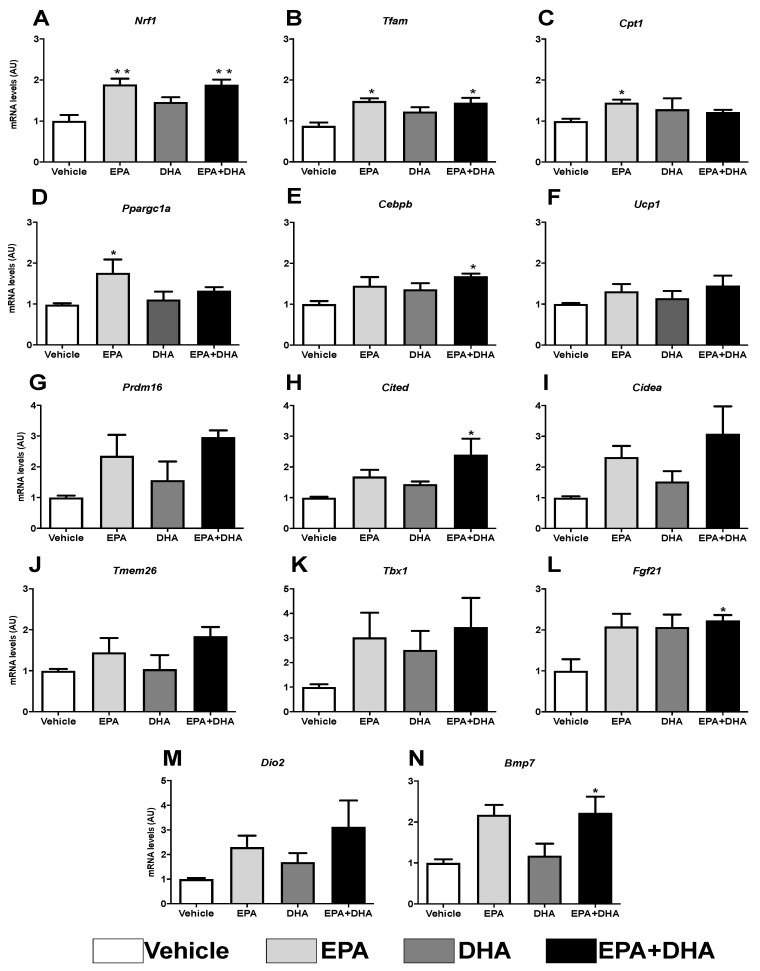
Evaluation of gene expression of beige adipocyte markers after browning induction six days after differentiation into 3T3-L1 preadipocytes by RT-PCR in the presence or not (control) of 50 µM of eicosapentaenoic acid (EPA, C20:5 n-3), docosaexaenoic (DHA, C22:6 n-3) or their association in a 5:1 ratio (42 µM EPA: 8 µM DHA). (**A**) *Nrf1*; (**B**) *Tfam*; (**C**) *Cpt1*; (**D**) *Ppargc1a* (Pgc1-α); (**E**) *Cebpβ*; (**F**) *Ucp1*; (**G**) *Prdm16*; *(***H**) *Cited*; (**I**) *Cidea*; (**J**) *Tmem26*; (**K**) *Tbx*; (**L**) *Fgf21*; (**M**) *Dio2*; (**N**) *Bmp7*. Values of mRNA were expressed in relation to the control and corrected by the expression of the constitutive gene *36B4*. Values expressed as mean ± SEM (n = 4–7). * *p* < 0.05 vs. Control, ** *p* < 0.01 vs. Control. One-way ANOVA and Tukey post-test.

The results showed that, when compared to the control group (cocktail), the presence of 50 µM of EPA, alone or in association with DHA, promoted an increase of ~47% in the expression of the gene encoding nuclear respiratory factor (*Nrf-1*) (Figure 7A), as well as an increase of ~40% in the gene expression of mitochondrial transcription factor A (*Tfam*) (Figure 7B). DHA alone was not able to elicit any significant effect in these genes’ expression. In addition, it was observed that EPA, compared to control, induced a ~31% increase in the expression of the gene encoding carnitine palmitoyltransferase I (*Cpt*-1) (Figure 7C), which plays an important role in the mitochondrial oxidation of fatty acids, as well as an increased expression of genes that encode thermogenesis-related proteins, such as *Ucp1* (31%), *Prdm16* (57%), *Cited* (40%), *Cidea* (56%), *Tmem26* (30%), *Tbx1* (66%), *Fgf21* (51%), *Dio2* (66%), and *Bmp7* (53%) (Figure 7F–N).

These transcription factors are controlled by transcriptional co-activators, as is the case of the PPARG Coactivator 1 Alpha (*Pgc-1α*), which also showed a ~44% increase in transcription (Figure 7D).

Curiously, *Cebpα*, known as the transcriptional partner of *Pparγ* orchestrating differentiation of white adipocytes, is not necessary for the differentiation of brown adipocytes. On the other hand, the expression of the gene coding *Cebpβ* induces the expression of *Pgc1*-*α* in beige adipocytes, but not in white ones. Herein, it was observed that, in the presence of these same PUFAs (EPA and its association), there was an increase (31% and 41%, respectively) in *Cebpβ* transcription (Figure 7E).

## 4. Discussion

Obesity is a chronic disease and is defined as a disproportion between body mass and height, with an abnormal or excessive accumulation of lipids in WAT [1,2]. However, the literature reports an important distinction between a pathologic WAT expansion and a healthy WAT expansion in obesity. The pathologic WAT expansion is the rapid growth of fat mass through hypertrophy of existing fat cells (hypertrophic obesity), with a high degree of infiltration mainly of M1 macrophages (which leads to inflammation), limited development of blood vessels, and massive fibrosis (from hypoxia) [31]. Hypertrophied adipocytes become highly inflamed and lipolytic, being responsible for the elevated levels of free fatty acids (FFA) and increased deposition in other organs such as the heart and liver (lipotoxic effects) [3]. On the other hand, healthy expansion occurs by adipocyte hyperplasia (through de novo differentiation from progenitor cells) [32], along with the recruitment of other types of stromal cells, within appropriate angiogenesis, vascular, and extracellular matrix remodeling, and minimal inflammation. 

Adipocyte differentiation is now accepted to be a potent strategy to prevent hypertrophic obesity (an independent risk factor for type 2 diabetes) and a driver for healthy WAT expansion, while adipogenesis in subcutaneous stromal cells is markedly reduced in hypertrophic obesity [33]. In agreement, it was demonstrated that subcutaneous adipocyte precursor cells failure to recruit and differentiate is the cause of hypertrophic obesity [34,35]. The present study was selected to explore the ability of n-3 PUFAs to neutralize metabolic consequences of obesity using obese mice (by high-fat diet) treated with FO, as well as AdSCs extracted from these mice and induced in vitro differentiation.

It is postulated that the use of a diet rich in saturated and monounsaturated FAs in animal models causes metabolic changes characteristic of obesity [36]. Recent studies also have indicated that HFD is directly related to several secondary disorders, such as DM2, hypertension, dyslipidemia, and NASH [37]. Thus, since lard and vegetable fat are rich in saturated and monounsaturated FAs, respectively, they are used in studies to effectively cause metabolic changes [14,38,39,40]. For the preparation of HFD in the present study, were used lard, which contains 40% saturated FAs (24% palmitic) and 59% unsaturated FAs (44% oleic acid) and soybean oil, which contains 81% unsaturated (24% oleic and 54% linoleic acid) as a source of lipids (9:1 ratio), which represents 59% of the energy in the diet.

The development of NASH is associated with excessive consumption of saturated fat, which can result in hyperlipidemia and subsequent accumulation of liver lipids [41]. Long-term HFD (like the one employed in the present study) results in the emergence of NASH [42]. It is well known that the main function of PPARα is to control lipid metabolism, and its activation can increase the utilization and decrease the synthesis of lipids by modulating gene expressions of liver lipogenic proteins [43], including protein-1c sterol regulatory element binder (SREBP-1c), fatty acid synthase (FAS), diacylglycerol acyltransferase (DGAT) and carnitine palmitoyltransferase-1A (CPT-1A) [44]. Therefore, PPARα is considered a potential therapeutic target for hyperlipidemia in NASH [45].

Furthermore, it is known that n-3 PUFAs can bind and/or regulate receptors activated by peroxisome proliferators (PPARs), a mechanism that may explain part of the effects demonstrated here, where treatment with FO significantly reduced plasma levels of VLDL, AST, ALT, alkaline phosphatase, gamma GT, bilirubin, as well as the lipid content and liver weight of obese animals induced by HFD. The anti-inflammatory properties of n-3 PUFAs are postulated to reduce liver inflammation associated with hepatic steatosis [46]. The parallel changes in the total liver mass and its lipid content found in our study support the evidence that FO reduces the deposition of hepatic lipids.

Regarding dyslipidemia and hyperglycemia triggered by HFD, treatment with FO during the last eight weeks (simultaneously with the HFD diet) also completely reversed changes in the plasma glucose profile (decreased fasting glucose) and lipid (decreased TAG, total cholesterol, LDL, and increased HDL). Dyslipidemia is frequently seen in obese and/or diabetics and a plasma concentration of total and LDL cholesterol is associated with an increased risk of heart disease. The effects reported above observed by treatment with FO were already expected, seen by several studies in the literature, demonstrating that it reduces the consequences of obesity, such as dyslipidemia and insulin resistance [47].

The consumption of n-3 PUFAs has important positive effects on the body, such as neurological development [48], during pregnancy and lactation [49], cardiovascular and mental health [50], cell membrane structure and signal transduction, and facilitating glucose uptake by endothelial cells in the brain, as well as reducing the accumulation of body fat, dyslipidemia, and improvement in insulin resistance [51]. It is also known that EPA triggers the reduction of blood clotting and blood flow. DHA has anti-inflammatory effects [52] and elevates neuroprotective termed neuroprotectins [53].

In addition, studies carried out previously by our group demonstrated a potent beneficial action of FO on adipocytes isolated from WAT of HFD-induced obesity, preventing and treating the metabolic and endocrine dysfunctions of these cells triggered by the diet, in addition to an anti-obesogenic effect [13,14]. In humans, n-3 PUFAs intake is directly related to the presence of healthier metabolic profiles, making them able to assist in the treatment and prevention of obesity comorbidities, especially by improving the individual components of the metabolic syndrome [54].

The loss of body mass through lifestyle changes is the main strategy to combat obesity and obesity-related diseases [55], even though it is difficult to adhere to, and adjuvant treatments include pharmaceutical products [56], surgery [57], and dietary supplements [58]. Despite these agents, the prevalence of obesity continues to rise. Hence, alternatives to assist in weight loss and reduce fat depots are highly necessary. Natural bioactive, such as n-3 PUFAs, usually do not have adverse effects and may be safer than other modalities for the treatment of obesity. In studies with rodents, the n-3 PUFAs prevented the gain of body mass from the high-fat diet, with the majority of studies with supplementation concomitant to the induction of obesity [59] or earlier, in a prevention model [14]. In the present study, mice were fed an HFD for 16 weeks; the diet was efficient causing obesity after 8 weeks—the period I. Thus, to treat obesity, in the following eight weeks (period II), the animals were supplemented with FO. Although the treatment did not result in differences in food intake and caloric intake, it was able to partially reduce the animals’ body mass. 

Compared to CO, the HFD group shows an increase in body mass which corresponds to the adiposity (ingWAT was significantly higher), and the FO reversed this increase. This result corroborates with other papers [60,61,62].

The adipose organ is extremely plastic, as it can change size in response to environmental stimuli, such as energy overload/deficiency or temperature changes. This is attributed to two mechanisms, named hypertrophy and hyperplasia. It has been reported that, after feeding with HFD, subcutaneous WAT grows its size mainly due to hypertrophy, whereas, in visceral WAT, the increase in size is, at least in part, due to hyperplasia [63]. This demonstrates that environmental stimuli can differentially affect the recruitment of new cells based on the anatomical location of adipose tissue, further accentuating the plasticity of this organ. The balance between hypertrophy and hyperplasia is strongly affected by the metabolic state of obesity.

Smaller adipocytes retain their insulin sensitivity, while hypertrophied adipocytes are likely to become insulin resistant and secrete proinflammatory cytokines [64], such as tumor necrosis factor-alpha (TNFα) and interleukin 6 (IL-6), which can also induce insulin resistance in other tissues [65]. In addition, adipose tissue with hypertrophied adipocytes is less vascularized and hypoxic, which can lead to high levels of angiogenic factors, which in turn can accelerate fibrosis and inflammation [66].

A high concentration of n-3 PUFAs in human subcutaneous WAT has been reported to correlate with shrink size of adipocytes, contrasting with an increase in the concentration of saturated PUFAs, which leads to a growth of fat cells [67]. In the present study, there was a significant increase in both the mass of the WAT deposit and the volume of the isolated adipocyte (hypertrophy) of the HFD group when compared to the CO group. However, FO treatment partially reversed these effects. Additionally, FO treatment caused a significant increase in the cellularity of this pad.

A frequent and distinctive feature of metabolically healthy obese patients is the presence of smaller and more numerous adipocytes compared to obese with metabolic impairment, whereas, in rodent models, artificially formation of new adipocytes improved insulin sensitivity [68]. These findings suggest that de novo differentiation of adipocytes from pre-adipocytes (adipogenesis) has a beneficial effect to protect from metabolic impairment induced by obesity, highlighting both the importance of adipocyte tissue plasticity and higher amounts of subcutaneous WAT than visceral WAT are associated with healthier phenotype [69].

When evaluating the effect of in vivo FO treatment on the cell proliferation of primary mouse pre-adipocytes, we observed that, compared to the CO and HFD groups, AdSCs from the FO group showed an increase in the proliferation potential by 27% and 22%, respectively. Corroborating this finding, when we cultured the primary pre-adipocytes from the eutrophic mice (or pre-adipocytes of the 3T3-L1 lineage) treated in vitro with n-3 fatty acids (EPA and DHA alone, or in a 5:1 ratio, which associated simulates the concentration of n-3 PUFAs in FO), we also observed an increase in the proliferation potential in cells treated with EPA or combination (but not with isolated DHA).

As mentioned, an increase in the number of adipocytes due to an adipogenesis stimulus is associated with a smaller size of adipocytes and a metabolically healthy phenotype. Additionally, it is known of great importance and the large involvement of WAT in the maintenance of metabolic homeostasis, since this tissue promotes several effects in metabolism and has an endocrine function. Hence, investigating the in vivo effect of n-3 PUFAs on the proliferation potential of pre-adipocytes and on the recruitment of new adipocytes by adipogenesis stimulus constituted an important objective of the present work.

Previous reports have also shown that cells derived from the WAT SVF from mice and humans can differentiate into several lineages, including adipocytes, in primary cell culture. These multipotent cells have been named adipocyte-derived stromal (or stem) cells (ADSCs), which contain a population of cells that can undergo proliferation and differentiation to form new adipocytes, leading to WAT remodeling [70]. These cells can differentiate into adipose cells with a white or beige phenotypic profile, with remarkable consequences on the energy metabolism of these cells and, consequently, with repercussions on the energy metabolism of the organism [71].

After differentiation, these cells acquire the machinery necessary for lipid metabolism, lose their potential for proliferation, increase their insulin sensitivity, and secrete growth factors and other cytokines [72]. Differentiation of pre-adipocytes can be induced by the addition of specific cocktails containing growth factors or *PPARγ* agonists [73], *PPARγ* being considered the main regulator of adipogenesis [74,75]. It is important to mention that PUFAs are their natural ligands [76]. It has been reported that in vitro both n-3 PUFAs (EPA and DHA) can bind and regulate *PPARγ* to induce differentiation of adipose cells and accelerate maturation, raising the expression of lipoprotein lipase [77].

In our study, after inducing differentiation, the primary adipocytes from the HFD group showed a 20% reduction in their lipid content, when compared to the CO group. The treatment of the animal with FO changed AdSCs capacity in accumulating lipids after in vitro differentiation, suggesting an important role of in vivo treatment with FO on AdSCs adipogenic potential. Thus, herein we demonstrates for the first time that the effects of FO treatment extend to non-differentiated SVF cells and persist after successive mitosis during proliferation.

Accordingly, in vitro treatment of AdSCs (extracted from eutrophic mice, not induced to obesity and untreated) since the differentiation induction day (day 0) with the n-3 PUFA EPA, DHA, and association (EPA/DHA) promoted an increase in the cell’s lipid content, suggesting an improvement in adipogenesis.

Taken together, we conclude that the treatment of obese animals with FO is effective in decreasing body mass, dyslipidemia, and NASH, as well as the adiposity of ingWAT and the hypertrophy of its adipocytes, besides promoting an increase in the cellularity of this depot. Adding these to the data obtained with AdSCs, we suggest that n-3 PUFAs might exert a stimulatory effect on the proliferation and differentiation of pre-adipocytes and, thus, might promote the recruitment of new adipocytes (white and/or initiated) in ingWAT, which is fundamental to the expansion of healthy tissue, capable of dealing with excess lipids offered by the diet. 

## Figures and Tables

**Table 1:** Sense and antisense primer sequences used for real-time quantitative PCR.

Gene	5′ Primer (5′-3′)—*Sense*	3′ Primer (5′-3′)—*Antisense*
*36B4*	TAAAGACTGGAGACAAGGTG	GTGTACTCAGTCTCCACAGA
*18S*	GAGAGGGAGCCTGAGAAAC	GGCCTGCTTTGAACACTC
*Lipe*	GGGAGGGCCTCAGCGTTCTCACA	ATAGCACGGAGCTGGGTGAGGG
*Fabp4*	AAGGTGAAGAGCATCATAACCCT	TCACGCCTTTCATAACACATTCC
*Slc2a4*	CATTCCCTGGTTCATTGTGG	GAAGACGTAAGGACCCATAGC
*AdipoQ*	GCAGAGATGGCACTCCTGGA	CCCTTCAGCTCCTGTCATTCC
*Cebpa*	CGCAAGAGCCGAGATAAAGC	CAGTTCACGGCTCAGCTGTTC
*Pnpla2*	GGTCCTCTGCATCCCTCCTT	CTGTCCTGAGGGAGATGTC
*Lpl*	GGCCAGATTCATCAACTGGAT	GCTCCAAGGCTGTACCCTAAG
*Fasn*	AGAGGCTTGTGCTGACTTCC	GTGGCTTCGGCGATGAGAG
*Acaca*	GAGAGGGGTCAAGTCCTTCC	ACATCCACTTCCACACACGA
*Dgat1*	AGAGGTGTTGGGAGGATCTG	GTCTGAGTGGGTGGCAGGT
*Lpin1*	TGATGTGGTGTTCAGTGTCACT	TCGTTGACCCAGTGCAGGTA
*Nrf1*	CGCAGCACCTTTGGAGAA	CCCGACCTGTGGAATACTTG
*Pparg2*	GCATCAGGCTTCCACTATGGA	AAGGCACTTCTGAAACCGACA
*Cebpb*	GCAAGAGCCGCGACAAG	GGCTCGGGCAGCTGCTT
*Ppargc1a*	ATCTACTGCCTGGGGACCTT	ATGTGTCGCCTTCTTGCTCT
*Tfam*	GGAATGTGGAGCGTGCTAAAA	TGCTGGAAAAACACTTCGGAATA
*Ucp1*	CACCTTCCCGCTGGACACT	CCCTAGGACACCTTTATACCTAATGG
*Prdm16*	CCACCAGCGAGGACTTCAC	GGAGGACTCTCGTAGCTCGAA
*Cidea*	ATCACAACTGGCCTGGTTACG	TACTACCCGGTGTCCATTTCT
*Cited1*	CGCTTCGTCCGTACCTCAGCT	CAGCTGGGCCTGTTGGTCTC
*Tmem26*	GAAACCAGTATTGCAGCACCC	CCAGACCGGTTCACATACCA
*Tbx1*	CGAATGTTCCCCACGTTCCA	GTGTACTCGGCCAGGTGTAG
*Fgf21*	CGTCTGCCTCAGAAGGACTC	TCTACCATGCTCAGGGGGTC
*Dio2*	AATTATGCCTCGGAGAAGACCG	GGCAGTTGCCTAGTGAAAGGT
*Pparalfa*	TCGGACTCGGTCTTCTTGAT	TCTTCCCAAAGCTCCTTCAA
*Bmp7*	CCTGTCCATCTTAGGGTTGC	GCCTTGTAGGGGTAGGAGAAG
*Srebf1*	CACTCCCTCTGATGCTACGG	CTTGTTTGCGATGTCTCCAG
*Cpt1*	TGTCCAAGTATCTGGCAGTCG	CATAGCCGTCATCAGCAACC

*36B4*: Acidic ribosomal phosphoprotein P0; *18S*: 18 S ribosomal RNA; *Lipe* (*Hsl*): hormone sensitive type; *Fabp4*: Fatty Acid Binding Protein 4; *Slc2a4* (*Glut-4*): glucose transporter 4; *AdipoQ*: adiponectin; *Cebpa* (*C/EBP-α*): CCAAT/enhancer-binding protein alpha; *Pnpal2* (*Atgl*): adipocyte triacylglycerol lipase; *Lpl*: Lipoprotein lipase; *Fasn*: fatty acid sinthase; *Acaca* (*Acc1*): Acetyl-CoA carboxylase; *Dgat1*: Diacylglycerol acyltransferase 1; *Lpin1*: lipin 1; *Nrf1*: Nuclear Respiratory Factor 1; *Pparg2* (*Ppar-γ2*): Peroxisome proliferator-activated receptor gamma; *Cebpb* (*C/EBP-β*): CCAAT/enhancer-binding protein beta; *Ppargc1a* (*Pgc1-α*): Peroxisome Proliferator-Activated Receptor Gamma Coativator 1-alpha; *Tfam*: Transcription Factor A, Mitochondrial; *Ucp1*: Uncoupler protein 1; *Prdm16*: PR-domain containing 16; Cidea: Cell Death Inducing DFFA Like Effector A; *Cited1*: Cbp/P300 Interacting Transactivator With Glu/Asp Rich Carboxy-Terminal Domain 1; *Tmem26*: Transmembrane Protein 26; *Tbx1*: T-Box Transcription Factor 1; *Fgf21*: Fibroblast Growth Factor 21; *Dio2*: Iodothyronine Deiodinase 2; *Pparalfa*: Peroxisome Proliferator Activated Receptor Alpha; *Bmp7*: Bone Morphogenetic Protein 7; *Srebf1*: Sterol Regulatory Element Binding Transcription Factor 1; *Cpt1*: carnitine palmitoyltransferase 1.

## Data Availability

The data presented in this study is contained within the article and available on request from the corresponding author.

## References

[1] Chechi K., Nedergaard J., Richard D. (2014). Brown adipose tissue as an anti-obesity tissue in humans. Obes. Rev..

[2] González-Muniesa P., Mártinez-González M.A., Hu F.B., Després J.P., Matsuzawa Y., Loos R.J.F., Moreno L.A., Bray G.A., Martinez J.A. (2017). Obesity. Nat. Rev. Dis. Prim..

[3] Albuquerque D., Stice E., Rodríguez-López R., Manco L., Nóbrega C. (2015). Current review of genetics of human obesity: From molecular mechanisms to an evolutionary perspective. Mol. Genet. Genom..

[4] Than N.N., Newsome P.N. (2015). A concise review of non-alcoholic fatty liver disease. Atherosclerosis.

[5] Xiang Z., Chen Y., Ma K., Ye Y., Zheng L., Li Y., Jin X. (2013). The role of ursodeoxycholic acid in non-alcoholic steatohepatitis: A systematic review. BMC Gastroenterol..

[6] Pagadala M., Kasumov T., McCullough A.J., Zein N.N., Kirwan J.P. (2012). Role of ceramides in nonalcoholic fatty liver disease. Trends Endocrinol. Metab..

[7] Angelico F., Del Ben M., Conti R., Francioso S., Feole K., Fiorello S., Cavallo M.G., Zalunardo B., Lirussi F., Alessandri C. (2005). Insulin resistance, the metabolic syndrome, and nonalcoholic fatty liver disease. J. Clin. Endocrinol. Metab..

[8] Adams L.A., Anstee Q.M., Tilg H., Targher G. (2017). Non-alcoholic fatty liver disease and its relationship with cardiovascular disease and other extrahepatic diseases. Gut.

[9] World Health Organization Obesity. https://www.who.int/health-topics/obesity#tab=tab_1.

[10] Anstee Q.M., Reeves H.L., Kotsiliti E., Govaere O., Heikenwalder M. (2019). From NASH to HCC: Current concepts and future challenges. Nat. Rev. Gastroenterol. Hepatol..

[11] Younossi Z.M., Koenig A.B., Abdelatif D., Fazel Y., Henry L., Wymer M. (2016). Global epidemiology of nonalcoholic fatty liver disease—meta-analytic assessment of prevalence, incidence, and outcomes. Hepatology.

[12] Browning J.D., Horton J.D. (2004). Molecular mediators of hepatic steatosis and liver injury. J. Clin. Investig..

[13] Mendez-Sanchez N., Cruz-Ramon V.C., Ramirez-Perez O.L., Hwang J.P., Barranco-Fragoso B., Cordova-Gallardo J. (2018). New aspects of lipotoxicity in nonalcoholic steatohepatitis. Int. J. Mol. Sci..

[14] Da Cunha de Sá R.D.C., Crisma A.R., Cruz M.M., Martins A.R., Masi L.N., do Amaral C.L., Curi R., Alonso-Vale M.I.C. (2016). Fish oil prevents changes induced by a high-fat diet on metabolism and adipokine secretion in mice subcutaneous and visceral adipocytes. J. Physiol..

[15] Da Cunha de Sá R.D.C., Cruz M.M., de Farias T.M., da Silva V.S., de Jesus Simão J., Telles M.M., Alonso-Vale M.I.C. (2020). Fish oil reverses metabolic syndrome, adipocyte dysfunction, and altered adipokines secretion triggered by high-fat diet-induced obesity. Physiol. Rep..

[16] Berná G., Romero-Gomez M. (2020). The role of nutrition in non-alcoholic fatty liver disease: Pathophysiology and management. Liver Int..

[17] Abenavoli L., Milanović M., Milić N., Luzza F., Giuffrè A.M. (2019). Olive oil antioxidants and non-alcoholic fatty liver disease. Expert Rev. Gastroenterol. Hepatol..

[18] Abenavoli L., Boccuto L., Federico A., Dallio M., Loguercio C., Di Renzo L., De Lorenzo A. (2019). Diet and Non-Alcoholic Fatty Liver Disease: The Mediterranean Way. Int. J. Environ. Res. Public Health.

[19] Reagan-Shaw S., Nihal M., Ahmad N. (2008). Dose translation from animal to human studies revisited. FASEB J..

[20] Couet C., Delarue J., Ritz P., Antoine J.M., Lamisse F. (1997). Effect of dietary fish oil on body fat mass and basal fat oxidation in healthy adults. Int. J. Obes..

[21] Do Amaral C.L., Milagro F.I., Curi R., Martínez J.A. (2014). DNA methylation pattern in overweight women under an energy-restricted diet supplemented with fish oil. BioMed Res. Int..

[22] Bucolo G., David H. (1973). Quantitative determination of serum triglycerides by the use of enzymes. Clin. Chem..

[23] Postiglione A., Cicerano U., Gallotta G., Gnasso A., Lamenza F., Rubba P., Mancini M. (1992). Prevalence of peripheral arterial disease and related risk factors in elderly institutionalized subjects. Gerontology.

[24] Grillo F., Izzo C., Mazzotti G., Murador E. (1981). Improved method for determination of high-density-lipoprotein cholesterol II. Enzymic determination of cholesterol in high-density lipoprotein fractions with a sensitive reagent. Clin. Chem..

[25] Bancroft J.D., Gamble M. (2008). Theory and Practice of Histological Techniques.

[26] Fischer A.H., Jacobson K.A., Rose J., Zeller R. (2008). Hematoxylin and eosin staining of tissue and cell sections. Cold Spring Harb. Protoc..

[27] Catta-Preta M., Mendonca L.S., Fraulob-Aquino J., Aguila M.B., Mandarim-de-Lacerda C.A. (2011). A critical analysis of three quantitative methods of assessment of hepatic steatosis in liver biopsies. Virchows Arch..

[28] Rodbell M. (1964). Metabolism of Isolated Fat Cells: I. Effects of Hormones on Glucose metabolism and lipolysis. J. Biol. Chem..

[29] Seo J.-H., Moon H.-S., Kim I.-Y., Guo D.-D., Lee H.-G., Choi Y.-J., Cho C.-S. (2008). PEGylated conjugated linoleic acid stimulation of apoptosis via a p53-mediated signaling pathway in MCF-7 breast cancer cells. Eur. J. Pharm. Biopharm..

[30] Denizot F., Lang R. (1986). Rapid colorimetric assay for cell growth and survival: Modifications to the tetrazolium dye procedure giving improved sensitivity and reliability. J. Immunol. Methods.

[31] Sun K., Kusminski C.M., Scherer P.E. (2011). Adipose tissue remodeling and obesity. J. Clin. Investig..

[32] Shao M., Vishvanath L., Busbuso N.C., Hepler C., Shan B., Sharma A.X., Chen S., Yu X., An Y.A., Zhu Y. (2018). De novo adipocyte differentiation from Pdgfrβ+ preadipocytes protects against pathologic visceral adipose expansion in obesity. Nat. Commun..

[33] Gustafson B., Hammarstedt A., Hedjazifar S., Smith U. (2013). Restricted adipogenesis in hypertrophic obesity: The role of WISP2, WNT, and BMP4. Diabetes.

[34] Arner P., Arner E., Hammarstedt A., Smith U. (2011). Genetic predisposition for Type 2 diabetes, but not for overweight/obesity, is associated with a restricted adipogenesis. PLoS ONE.

[35] Jansson P.-A., Pellmé F., Hammarstedt A., Sandqvist M., Brekke H., Caidahl K., Forsberg M., Volkmann R., Carvalho E., Funahashi T. (2003). A novel cellular marker of insulin resistance and early atherosclerosis in humans is related to impaired fat cell differentiation and low adiponectin. FASEB J..

[36] Newmark H.L., Lipkin M. (2018). Colonic Hyperplasia and Hyperproliferation Induced in Rodents by a Nutritional Stress Diet Containing 4 Factors of The Western Human Diet: High Fat and Phosphate, Low Calcium and Vitamin D. Calcium, Vitamin D, and Prevention of Colon Cancer.

[37] Tabarés Seisdedos R. (2017). Health effects of overweight and obesity in 195 countries over 25 years. N. Engl. J. Med..

[38] Orsavova J., Misurcova L., Ambrozova J., Vicha R., Mlcek J. (2015). Fatty Acids Composition of Vegetable Oils and Its Contribution to Dietary Energy Intake and Dependence of Cardiovascular Mortality on Dietary Intake of Fatty Acids. Int. J. Mol. Sci..

[39] Ikemoto S., Takahashi M., Tsunoda N., Maruyama K., Itakura H., Ezaki O. (1996). High-fat diet-induced hyperglycemia and obesity in mice: Differential effects of dietary oils. Metabolism.

[40] Rendina-Ruedy E., Smith B.J. (2016). Methodological considerations when studying the skeletal response to glucose intolerance using the diet-induced obesity model. Bonekey Rep..

[41] Foulds C.E., Treviño L.S., York B., Walker C.L. (2017). Endocrine-disrupting chemicals and fatty liver disease. Nat. Rev. Endocrinol..

[42] Yu D., Chen G., Pan M., Zhang J., He W., Liu Y., Nian X., Sheng L., Xu B. (2018). High fat diet-induced oxidative stress blocks hepatocyte nuclear factor 4α and leads to hepatic steatosis in mice. J. Cell. Physiol..

[43] Kersten S. (2014). Integrated physiology and systems biology of PPARα. Mol. Metab..

[44] Montagner A., Polizzi A., Fouché E., Ducheix S., Lippi Y., Lasserre F., Barquissau V., Régnier M., Lukowicz C., Benhamed F. (2016). Liver PPARα is crucial for whole-body fatty acid homeostasis and is protective against NAFLD. Gut.

[45] Zhao X., Wang F., Zhou R., Zhu Z., Xie M. (2018). PPARα/γ antagonists reverse the ameliorative effects of osthole on hepatic lipid metabolism and inflammatory response in steatohepatitic rats. Inflammopharmacology.

[46] Scorletti E., Byrne C.D. (2018). Omega-3 fatty acids and non-alcoholic fatty liver disease: Evidence of efficacy and mechanism of action. Mol. Aspects Med..

[47] Casula M., Soranna D., Catapano A.L., Corrao G. (2013). Long-term effect of high dose omega-3 fatty acid supplementation for secondary prevention of cardiovascular outcomes: A meta-analysis of randomized, double blind, placebo controlled trials. Atheroscler. Suppl..

[48] Meyer B.J., Onyiaodike C.C., Brown E.A., Jordan F., Murray H., Nibbs R.J.B., Sattar N., Lyall H., Nelson S.M., Freeman D.J. (2016). Maternal plasma DHA levels increase prior to 29 days post-LH surge in women undergoing frozen embryo transfer: A prospective, observational study of human pregnancy. J. Clin. Endocrinol. Metab..

[49] Makrides M., Gibson R.A. (2000). Long-chain polyunsaturated fatty acid requirements during pregnancy and lactation. Am. J. Clin. Nutr..

[50] Sinn N., Milte C., Howe P.R.C. (2010). Oiling the brain: A review of randomized controlled trials of omega-3 fatty acids in psychopathology across the lifespan. Nutrients.

[51] Flachs P., Rossmeisl M., Kopecky J. (2014). The effect of n-3 fatty acids on glucose homeostasis and insulin sensitivity. Physiol. Res..

[52] Duvall M.G., Levy B.D. (2016). DHA-and EPA-derived resolvins, protectins, and maresins in airway inflammation. Eur. J. Pharmacol..

[53] Parletta N., Milte C.M., Meyer B.J. (2013). Nutritional modulation of cognitive function and mental health. J. Nutr. Biochem..

[54] Siriwardhana N., Kalupahana N.S., Moustaid-Moussa N. (2012). Health benefits of n-3 polyunsaturated fatty acids: Eicosapentaenoic acid and docosahexaenoic acid. Advances in Food and Nutrition Research.

[55] Schakarowski F.B., Padoin A.V., Mottin C.C., Castro E.K. (2018). De Percepção de risco da cirurgia bariátrica em pacientes com diferentes comorbidades associadas à obesidade. Trends Psychol..

[56] Patel D. (2015). Pharmacotherapy for the management of obesity. Metabolism.

[57] Arterburn D.E., Courcoulas A.P. (2014). Bariatric surgery for obesity and metabolic conditions in adults. BMJ.

[58] Raynor H.A., Champagne C.M. (2016). Position of the Academy of Nutrition and Dietetics: Interventions for the Treatment of Overweight and Obesity in Adults. J. Acad. Nutr. Diet..

[59] Kleinert M., Clemmensen C., Hofmann S.M., Moore M.C., Renner S., Woods S.C., Huypens P., Beckers J., De Angelis M.H., Schürmann A. (2018). Animal models of obesity and diabetes mellitus. Nat. Rev. Endocrinol..

[60] Duivenvoorde L.P.M., van Schothorst E.M., Swarts H.M., Kuda O., Steenbergh E., Termeulen S., Kopecky J., Keijer J. (2015). A difference in fatty acid composition of isocaloric high-fat diets alters metabolic flexibility in male C57BL/6JOlaHsd mice. PLoS ONE.

[61] Bertrand C., Pignalosa A., Wanecq E., Rancoule C., Batut A., Deleruyelle S., Lionetti L., Valet P., Castan-Laurell I. (2013). Effects of Dietary Eicosapentaenoic Acid (EPA) Supplementation in High-Fat Fed Mice on Lipid Metabolism and Apelin/APJ System in Skeletal Muscle. PLoS ONE.

[62] Sato A., Kawano H., Notsu T., Ohta M., Nakakuki M., Mizuguchi K., Itoh M., Suganami T., Ogawa Y. (2010). Antiobesity effect of eicosapentaenoic acid in high-fat/high-sucrose diet–induced obesity: Importance of hepatic lipogenesis. Diabetes.

[63] Jeffery E., Church C.D., Holtrup B., Colman L., Rodeheffer M.S. (2015). Rapid depot-specific activation of adipocyte precursor cells at the onset of obesity. Nat. Cell Biol..

[64] Jernås M., Palming J., Sjöholm K., Jennische E., Svensson P.-A., Gabrielsson B.G., Levin M., Sjögren A., Rudemo M., Lystig T.C. (2006). Separation of human adipocytes by size: Hypertrophic fat cells display distinct gene expression. FASEB J..

[65] Chawla A., Nguyen K.D., Goh Y.P.S. (2011). Macrophage-mediated inflammation in metabolic disease. Nat. Rev. Immunol..

[66] Lee Y.S., Kim J., Osborne O., Sasik R., Schenk S., Chen A., Chung H., Murphy A., Watkins S.M., Quehenberger O. (2014). Increased adipocyte O2 consumption triggers HIF-1α, causing inflammation and insulin resistance in obesity. Cell.

[67] Garaulet M., Hernandez-Morante J.J., Lujan J., Tebar F.J., Zamora S. (2006). Relationship between fat cell size and number and fatty acid composition in adipose tissue from different fat depots in overweight/obese humans. Int. J. Obes..

[68] Kusminski C.M., Holland W.L., Sun K., Park J., Spurgin S.B., Lin Y., Askew G.R., Simcox J.A., McClain D.A., Li C. (2012). MitoNEET-driven alterations in adipocyte mitochondrial activity reveal a crucial adaptive process that preserves insulin sensitivity in obesity. Nat. Med..

[69] Pellegrinelli V., Carobbio S., Vidal-Puig A. (2016). Adipose tissue plasticity: How fat depots respond differently to pathophysiological cues. Diabetologia.

[70] Rodeheffer M.S., Birsoy K., Friedman J.M. (2008). Identification of white adipocyte progenitor cells in vivo. Cell.

[71] Villarroya F., Cereijo R., Villarroya J., Giralt M. (2017). Brown adipose tissue as a secretory organ. Nat. Rev. Endocrinol..

[72] Contador D., Ezquer F., Espinosa M., Arango-Rodriguez M., Puebla C., Sobrevia L., Conget P. (2015). Featured Article: Dexamethasone and rosiglitazone are sufficient and necessary for producing functional adipocytes from mesenchymal stem cells. Exp. Biol. Med..

[73] Bunnell B.A., Flaat M., Gagliardi C., Patel B., Ripoll C. (2008). Adipose-derived stem cells: Isolation, expansion and differentiation. Methods.

[74] Cohen P., Levy J.D., Zhang Y., Frontini A., Kolodin D.P., Svensson K.J., Lo J.C., Zeng X., Ye L., Khandekar M.J. (2014). Ablation of PRDM16 and beige adipose causes metabolic dysfunction and a subcutaneous to visceral fat switch. Cell.

[75] Tzameli I., Fang H., Ollero M., Shi H., Hamm J.K., Kievit P., Hollenberg A.N., Flier J.S. (2004). Regulated production of a peroxisome proliferator-activated receptor-γ ligand during an early phase of adipocyte differentiation in 3T3-L1 adipocytes. J. Biol. Chem..

[76] Madsen L., Petersen R.K., Kristiansen K. (2005). Regulation of adipocyte differentiation and function by polyunsaturated fatty acids. Biochim. Biophys. Acta BBA Mol. Basis Dis..

[77] Mater M.K., Pan D., Bergen W.G., Jump D.B. (1998). Arachidonic acid inhibits lipogenic gene expression in 3T3-L1 adipocytes through a prostanoid pathway. J. Lipid Res..

